# Effects of Vessel Interruption Sequence During Lobectomy for Non-small Cell Lung Cancer: A Systematic Review and Meta-Analysis

**DOI:** 10.3389/fsurg.2021.694005

**Published:** 2021-07-26

**Authors:** Xiang Long, Bingxuan Wu, Wenxiong Zhang, Guoli Lv, Dongliang Yu, Jinhua Peng, Yiping Wei, Youming Lei

**Affiliations:** ^1^Department of Cardio-Thoracic Surgery, The Second Affiliated Hospital of Nanchang University, Nanchang, China; ^2^Jiangxi Medical College, Nanchang University, Nanchang, China; ^3^Department of Thoracic Surgery, The First Affiliated Hospital of Kunming Medical University, Kunming, China

**Keywords:** vessel interruption sequence, non-small cell lung cancer, meta-analysis, lobectomy, systematic review

## Abstract

**Background:** For lobectomy in non-small cell lung cancer (NSCLC), whether interrupting the pulmonary vein first (Vein-first) achieves better perioperative and survival outcomes than interrupting the pulmonary artery first (Artery-first) remains controversial. We conducted this meta-analysis to compare outcomes between the two groups to facilitate better surgical decision-making.

**Methods:** Web of Science, EMBASE, Cochrane Library, Ovid MEDLINE, PubMed, ScienceDirect, and Scopus were searched for eligible studies comparing Vein-first and Artery-first procedures. The primary endpoints were survival indicators [overall survival (OS), disease-free survival (DFS), and lung cancer-specific survival (LCSS)]. Secondary endpoints included intraoperative indicators, hospitalization, and follow-up indicators.

**Results:** After screening 2,505 studies, 8 studies involving 1,714 patients (Vein-First group: 881 patients; Artery-first group: 833 patients) were included. The vein-first group achieved better OS [HR (hazard ratio): 1.46, 95% confidence interval (CI): 1.12–1.91, *p* = 0.005], DFS (HR: 1.60, 95% CI: 1.23–2.08, *p* < 0.001), and LCSS (HR: 1.64, 95% CI: 1.16–2.31, *p* = 0.005). The survival rates of OS at 2–5 years, DFS at 1–5 years, and LCSS at 3–5 years were also higher in the Vein-First group. Subgroup analyses suggested that the advantages of survival in the Vein-First group were primarily embodied in the subgroups of squamous cell carcinoma (SCC) and earlier pathological TNM stage (I–II). Operative time, intraoperative blood loss, total complications, and total recurrences were comparable between the two groups.

**Conclusions:** The Vein-first sequence is the suitable choice of vessel interruption sequence during lobectomy for NSCLC with better survival and similar perioperative outcomes, especially for stage I–II SCC.

## Introduction

In the past decade, lung cancer was the main cause of cancer-related death worldwide (1, 2). Lobectomy has been used for decades in clinical practice as a classical surgical procedure for stage I–IIIA non-small cell lung cancer (NSCLC) (3). Interruptions of the pulmonary artery (PA) and pulmonary vein (PV) are the essential procedures for lobectomy. However, the choice of which blood vessel to interrupt first is an easily neglected problem in practice (4).

The effects of the interruption sequence of PA and PV has been a long-debated issue, and currently, no guidelines have been confirmed (5, 6). Wei et al. compared 86 patients in a randomized clinical trial (RCT) and suggested that ligation of the effluent veins first reduced tumor cell dissemination and improved survival outcomes (7). He et al. and Sumitomo et al. also reported similar results that favored the pulmonary vein first (Vein-first) group, especially for squamous cell carcinoma (SCC) (8, 9). However, several studies showed that the two groups achieved similar long-term survival and postoperative recurrences (10–12). Li et al. suggested that pulmonary vein interruption first increased blood loss without affecting the operative difficulty, tumor recurrence, metastasis, or survival (13).

To clarify this controversy and standardize the surgical process for a better prognosis of patients with NSCLC, we compared the relation of Vein-first and pulmonary artery first (Artery-first) surgical techniques to perioperative and survival outcomes.

## Materials and Methods

This study was conducted following the Preferred Reporting Items for Systematic Reviews and Meta-Analyses (PRISMA) guidelines (Supplementary Table 1) (14).

### Search Strategy

Web of Science, EMBASE, Cochrane Library, Ovid MEDLINE, PubMed, ScienceDirect, and Scopus databases were systematically searched from inception to December 6, 2020, for studies analyzing the effects of vessel interruption sequence during thoracoscopic lobectomy for NSCLC. The following MeSH terms were used: “vein”, “artery,” and “lung cancer.” The references of the retrieved literature (including meta-analyses and abstracts), bibliographies and gray literatures were also searched for further eligible articles. The detailed retrieval strategies are shown in Supplementary Table 2.

#### Selection Criteria

Inclusion criteria:

(1) Population: patients with NSCLC who underwent lobectomy.
(2) Intervention and comparison: Vein-First sequence (the PVs in the hilum of pulmonary lobes were dissected and transected first) vs. Artery-First sequence (all pulmonary arteries were to be completely ligated before venous interruption).
(3) Outcomes: survival, intraoperative outcomes, hospitalization, and follow-up outcomes.
(4) Study design: RCTs or cohort studies.

We excluded pure basic studies, reviews, animal experiments, and articles lacking original data.

### Data Extraction

The following data were extracted by two independent investigators (XL and WXZ): the published year, first author, country, study period, participant characteristics (sex, age, comorbidity, and smoking status), tumor characteristics (histology, location, pathological stage), survival [overall survival (OS), disease-free survival (DFS), and lung cancer-specific survival (LCSS)], intraoperative outcomes (operative time, blood loss, and blood transfusion), hospitalization, and follow-up outcomes [postoperative hospital stay, postoperative drainage time, total complications, increment of circulating tumor cells (CTCs), and recurrences]. Any discrepancies between the investigators were resolved by a third author (YML).

#### Outcome Assessments

In addition to analyzing survival data (OS, PFS, and LCSS), we analyzed the survival rate at 1–5 years (OSR, PFSR, and LCSSR). We also analyzed the subgroup data of OS, DFS, and LCSS according to age, sex, comorbidity, smoking status, tumor location, sequence of vessel ligation, tumor size, N stage, pathological TNM stage, histological type, postoperative adjuvant therapy, use of a stapler, and type of resection.

### Quality Assessment for Included Studies

The Newcastle-Ottawa Scale (NOS) was used to assess the quality of cohort studies. The scale included three items: comparability, selection, and outcome. Scores ≥6 points indicate medium-high quality (15). A five-point Jadad scale was used to assess the quality of RCTs. The scale included three items: randomization, masking, and accountability of all patients. Scores ≥3 points indicate high quality (16).

The Grades of Recommendations Assessment, Development, and Evaluation (GRADE) system was used to assess the evidence level of the results. The system included five items: imprecision, risk of bias, indirectness, inconsistency, and publication bias. Very low, low, moderate, and high were the four levels of evidence (17).

### Statistical Analysis

Review Manager 5.3 (The Nordic Cochrane Centre, The Cochrane Collaboration, Copenhagen, Denmark) and STATA 12.0 (StataCorp, College Station, TX, USA) were used to analyze the pooling data. We used HRs to analyze the survival data (OS, DFS, and LCSS). When the HR > 1, then the results supported the Vein-First group. We used the difference in means (MD) to analyze the continuous variables (operative time, postoperative drainage time, and increment of CTCs). We used the pooled risk ratios (RRs) to analyze the dichotomous variables (OSR, PFSR, LCSSR, blood transfusion, total complications, recurrences, and rate of CTC increase). In the analysis of OSR, PFSR, and LCSSR, the results supported the Vein-First group when the RR > 1. In the analysis of other variables, the results supported the Vein-First group when the RR <1. The HRs of survival data were extracted directly from the seven studies or the Kaplan-Meier curves according to Tierney's method (18). The *I*^2^ statistic and χ^2^-test were used to assess the heterogeneity. The random-effects model was used for significant heterogeneity (*I*^2^ > 50% or *p* < 0.1). Otherwise, the fixed-effects model was used. Egger's (19) and Begg's tests (20) were used to assess the publication bias. *P* = 0.05 was set as the statistical boundary value, and *p* < 0.05 indicated statistical significance.

## Results

### Search Results and Quality Assessment of the Included Studies

**Figure 1:**
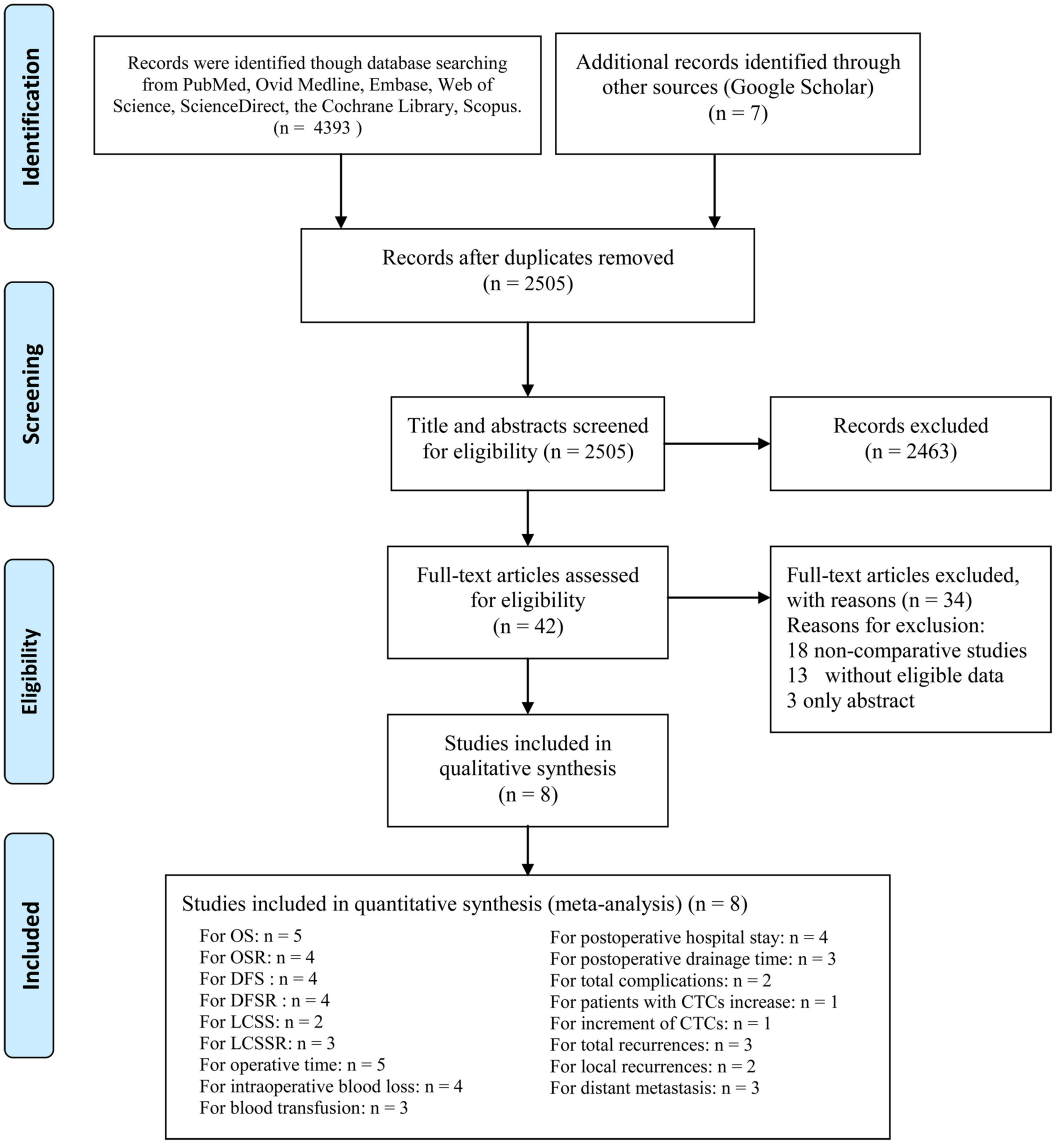
Flow chart of the study selection process.

A total of 2,505 studies were initially searched, and seven papers involving eight studies (Vein-First group: 881 patients; Artery-First group: 833 patients) were included for the final analysis (Figure 1) (7–13). Seven (7–9, 11–13) of the eight studies were conducted in Asia, and one (10) study was performed in Europe. Two studies were RCTs, and the other six studies were cohort studies. According to the NOS and Jadad scale, two studies (8, 9) were of medium quality, and six studies (7, 10–13) were of high quality (Supplementary Table 3). The baseline characteristics are listed in Table 1. According to the GRADE system, the quality evidence of all results were low to very low (Supplementary Table 4).

**Table 1:** Summary of the baseline characteristics of the included studies.

**Study**		**Country**	**Period (year)**	**Groups**	**Patients**	**Sex (M/F)**	**Age (Mean, year)**	**Lesion location (lobes)**					**Pathological TNM stage**^**a**^								**Follow up (months)**
							**Right**			**Left**		**I**		**II**		**III**		**IV**		
								**Upper**	**Middle**	**Lower**	**Upper**	**Lower**	**IA**	**IB**	**IIA**	**IIB**	**IIIA**	**IIIB**	**IVA**	**IVB**	
2019	Wei (7)-RCT	China	2016–2018	Vein-First	43	25/18	62.1	27			16		22		10		9		2		–
				Artery-First	43	26/17	63.2	28			15		21		10		12		0		
2019	Wei (7)-RT	China	2005–2017	Vein-First	210	113/97	59.7	139			71		137		33		40		0		30
				Artery-First	210	120/90	58.6	126			84		128		30		52		0		
2019	He (8)	China	2012–2013	Vein-First	33	22/11	59.6	18	5	2	8	0	8	6	2	6	10	0	1	0	54.5
				Artery-First	27	19/8	62.2	6	1	10	3	7	1	9	4	5	5	3	0	0	
2018	Sumitomo (9)	Japan	2007–2013	Vein-First	104	51/53	66.1	41	7	20	25	11	65	26	0	5	8	0	0	0	54.9
				Artery-First	83	41/42	66.2	13	6	31	14	19	55	15	3	5	5	0	0	0	
2015	Li (13)	China	2006–2013	Vein-First	174	94/80	62.8	76	27	17	41	13	138	36	0	0	30
				Artery-First	93	36/57	62.6	12	9	43	3	26	79	14	0	0	26
2013	Kozak (10)	Poland	1999–2003	Vein-First	170	124/46	60.2	–	–	–	–	–	76	24	39	0	62.4
				Artery-First	215	143/72	59.8	–	–	–	–	–	105	58	52	0	60.1
2007	Yellin (11)	Israel	2001–2003	Vein-First	14	8/6	66.6	6	0	3	4	1	–	–	–	–	–	–	–	–	–
				Artery-First	16	9/7	63.1	5	0	4	4	3	–	–	–	–	–	–	–	–	
2003	Refaely (12)	Israel	1992–1998	Vein-First	133	86/47	64.5	85			45		77		21		29		6		22.6
				Artery-First	146	89/57	65.7	78			68		75		29		39		3		

*M/F, male/female; TNM, tumor node metastasis*.

a *Pathological TNM stage: four studies [Wei (7)-RCT, Wei (7)-RT, He (8), and Sumitomo (9)] were according to 8th edition of TNM classification, two studies [Li (13) and Kozak (10)] were according to 7th edition of TNM classification, one studies [Refaely (12)] were according to 6th edition of TNM classification. Data on lung cancer staging are not available in Yellin (11)*.

#### Survival

**Figure 2:**
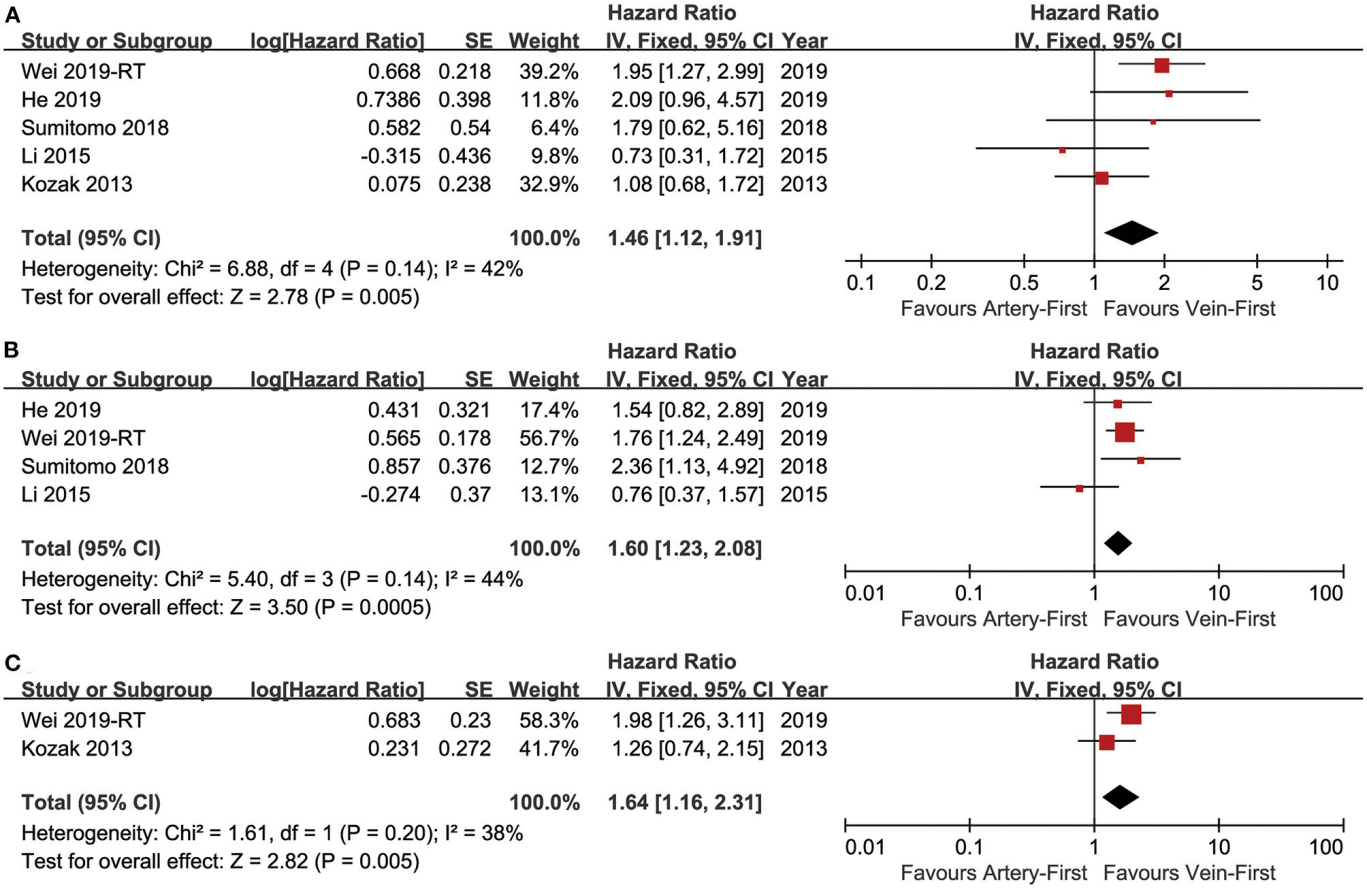
Forest plots of OS **(A)**, DFS **(B)**, and LCSS **(C)** associated with Vein-first vs. Artery-first.

**Figure 3:**
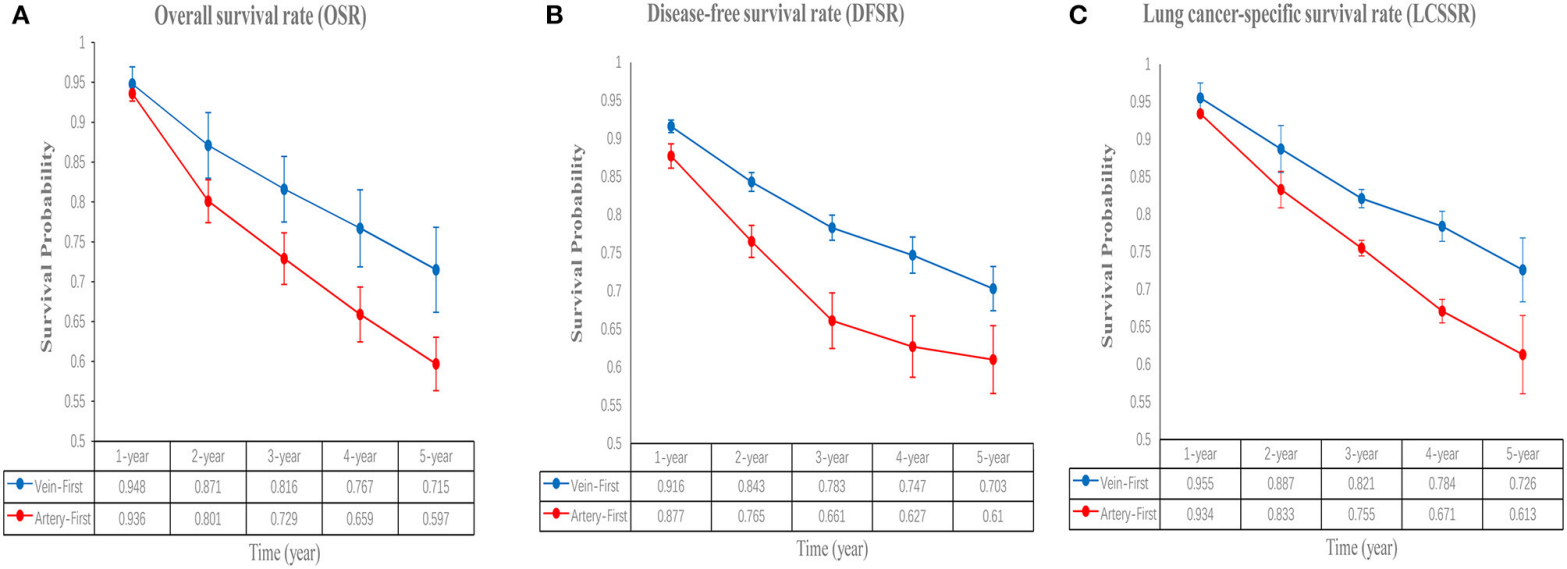
Comparisons of OSR (1–5 years, **A**), DFSR (1–5 years, **B**), and LCSSR (1–5 years, **C**) associated with Vein-first vs. Artery-first according to survival time.

**Figure 4:**
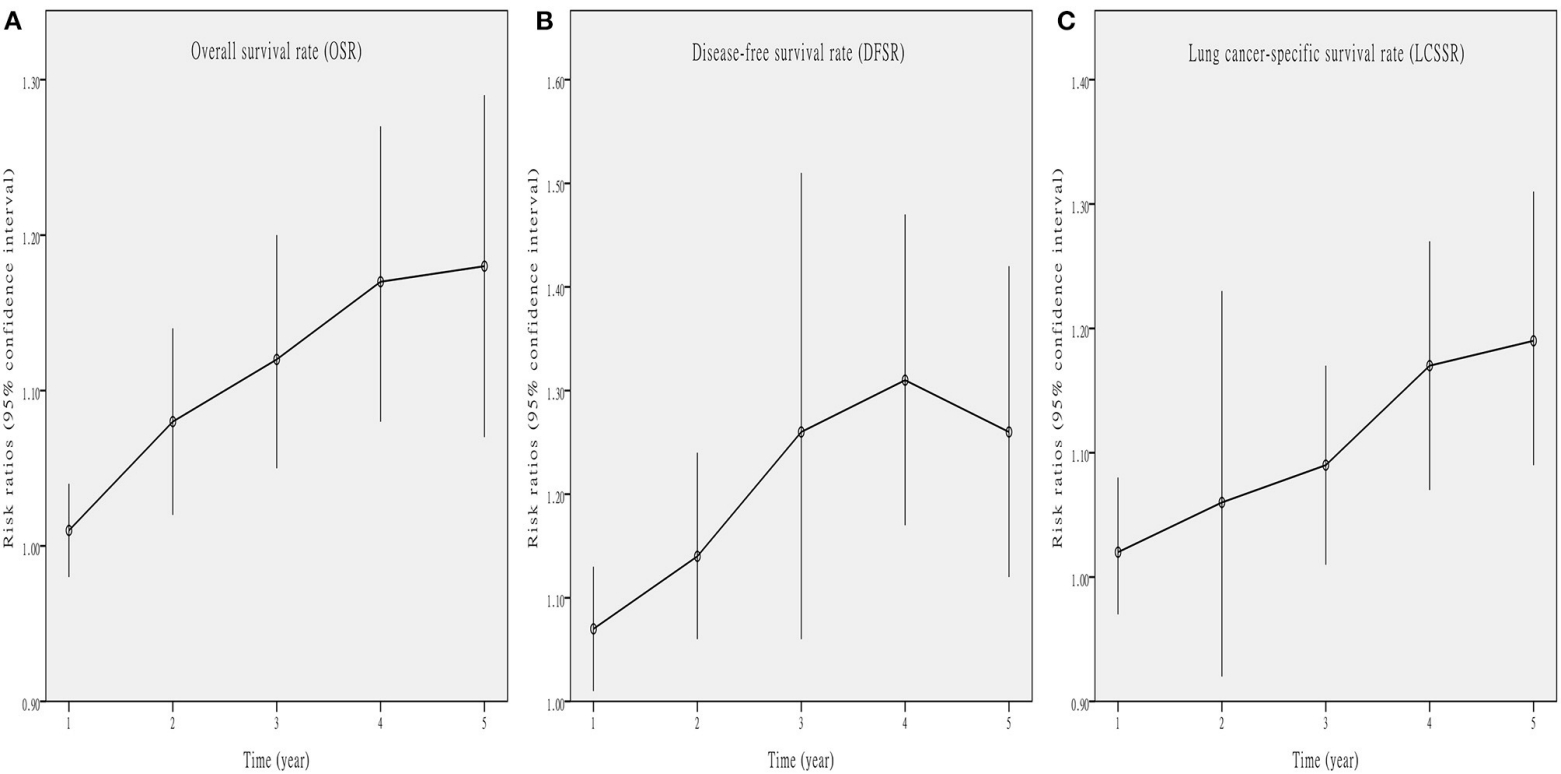
Line charts of OSR (1–5 years, **A**), DFSR (1–5 years, **B**), and LCSSR (1–5 years, **C**) associated with Vein-first vs. Artery-first according to survival time.

Five studies (7–10, 13) compared OS with acceptable heterogeneity (*I*^2^ = 42%). Better OS was found in the Vein-First group [HR: 1.46, 95% confidence interval (CI): 1.12–1.91, *p* = 0.005, Figure 2A]. Subgroup analyses suggested that the Vein-First group achieved better OSR-2y (RR: 1.08, 95% CI: 1.02–1.14, *p* = 0.007), OSR-3y (RR: 1.12, 95% CI: 1.05–1.20, *p* = 0.001), OSR-4y (RR: 1.17, 95% CI: 1.08–1.27, *p* < 0.001), and OSR-5y (RR: 1.18, 95% CI: 1.07–1.29, *p* < 0.001) (Supplementary Figure 1 and Figure 3A). The overall survival advantage of the Vein-First group increased over time (Figure 4A).

Four studies (7–9, 13) compared DFS with acceptable heterogeneity (*I*^2^ = 44%). Better DFS was found in the Vein-First group (HR: 1.60, 95% CI: 1.23–2.08, *p* < 0.001, Figure 2B). Subgroup analyses suggested that the Vein-First group achieved better DFSR-1y (RR: 1.07, 95% CI: 1.01–1.13, *p* = 0.01), DFSR-2y (RR: 1.14, 95% CI: 1.06–1.24, *p* = 0.001), DFSR-3y (RR: 1.26, 95% CI: 1.06–1.51, *p* = 0.009), DFSR-4y (RR: 1.31, 95% CI: 1.17–1.47, *p* < 0.001), and DFSR-5y (RR: 1.26, 95% CI: 1.12–1.42, *p* < 0.001) (Supplementary Figure 2 and Figure 3B). The disease-free survival advantage of the Vein-First group increased over time (Figure 4B).

Two studies (7, 13) compared LCSS with acceptable heterogeneity (*I*^2^ = 38%). Better LCSS was found in the Vein-First group (HR: 1.64, 95% CI: 1.16–2.31, *p* = 0.005, Figure 2C). Subgroup analyses of LCSSR suggested that the Vein-First group achieved better LCSSR-3y (RR: 1.09, 95% CI: 1.01–1.17, *p* = 0.02), LCSSR-4y (RR: 1.17, 95% CI: 1.07–1.27, *p* < 0.001), and LCSSR-5y (RR: 1.19, 95% CI: 1.09–1.31, *p* < 0.001) (Supplementary Figure 3 and Figure 3C). The lung cancer-specific survival advantage of the Vein-First group increased over time (Figure 4C).

### Subgroup Analysis of Survival

Based on the included studies, we analyzed the factors that might affect the survival effect of lobectomy for patients with NSCLC. The results suggested that younger age, vein-first sequence, smaller tumor size, earlier N stage, and earlier pathological TNM stage were the favorable factors associated with better survival. No significant differences were found in the subgroup analyses according to sex (female vs. male), comorbidity (no vs. yes), current or former smoking (no vs. yes), tumor location (left lung vs. right lung), histological type (adenocarcinoma vs. non-adenocarcinoma), postoperative adjuvant therapy (no vs. yes), stapler use (no vs. yes), and type of resection (lobectomy vs. pneumonectomy) (Table 2).

**Table 2:** Subgroup analysis of survival (OS, DFS, and LCSS) in NSCLC patients after lobectomy.

**Subgroups**	**No. of studies**	**Overall survival**		**No. of studies**	**Disease-free survival**		**No. of studies**	**Lung cancer-specific survival**	
		**HR (95% CI)**	***P***		**HR (95% CI)**	***P***		**HR (95% CI)**	***P***
**Age, year**									
<60 vs. >60	2	1.03 (1.00–1.05)	0.02	1	0.80 (0.57–1.14)	0.22	1	0.96 (0.62–1.49)	0.85
**Sex**									
Female vs. Male	2	1.20 (0.94–1.54)	0.15	2	1.03 (0.76–1.39)	0.85	1	1.02 (0.67–1.57)	0.92
**Comorbidity**									
No vs. yes	1	1.05 (0.69–1.61)	0.82	2	0.96 (0.69–1.32)	0.78	1	0.94 (0.60–1.47)	0.77
**Current or former smoking**									
No vs. yes	1	1.21 (0.81–1.82)	0.35	2	1.19 (0.61–2.35)	0.61	1	1.11 (0.68–1.63)	0.82
**Tumor location**									
Left lung vs. right lung	1	1.06 (0.70–1.60)	0.79	1	1.08 (0.76–1.53)	0.66	1	1.05 (0.68–1.63)	0.82
Right upper lobe vs. right middle lobe	–	–	–	1	1.08 (0.27–6.74)	0.71	–	–	–
Right upper lobe vs. right lower lobe	–	–	–	1	1.36 (0.84–6.00)	0.11	–	–	–
Right upper lobe vs. left upper lobe	–	–	–	1	2.25 (0.45–4.32)	0.57	–	–	–
Right upper lobe vs. left lower lobe	–	–	–	1	1.54 (0.47–5.04)	0.48	–	–	–
**Sequence of vessel ligation**									
Vein-first vs. Artery-first	5	1.46 (0.12–1.91)	0.005	4	1.60 (1.23–2.08)	<0.001	1	1.98 (1.26–3.11)	0.003
**Tumor size, cm**									
<3 vs. ≥3	2	1.53 (1.19–1.97)	0.001	1	1.81 (1.29–2.53)	0.001	1	1.79 (1.17–2.74)	0.008
**N stage**									
N0 vs. N1-2	1	1.64 (1.25–2.17)	<0.001	–	–	–	–	–	–
**Pathological TNM stage**									
I vs. II	1	2.23 (1.26–3.96)	0.006	1	1.75 (1.06–2.91)	0.03	1	2.82 (1.55–5.11)	0.001
I vs. III	1	4.02 (2.57–6.30)	<0.001	1	4.18 (2.90–6.00)	<0.001	1	5.07 (3.14–8.18)	<0.001
I vs. II–IIIA	–	–	–	1	4.07 (1.95–8.52)	<0.001	–	–	–
**Histological type**									
Adenocarcinoma vs. Nonadenocarcinoma	1	1.21 (0.78–1.87)	0.39	2	1.67 (0.56–5.01)	0.36	1	1.18 (0.75–1.87)	0.47
**Postoperative adjuvant therapy**									
No vs. yes	1	1.00 (0.66–1.50)	0.99	1	1.13 (0.81–1.57)	0.49	1	1.14 (0.75–1.75)	0.54
**Stapler use**									
No vs. Yes	1	1.04 (0.72–1.51)	0.82	–	–	–	–	–	–
**Type of resection**									
Lobectomy vs. pneumonectomy	1	1.06 (0.76–1.49)	0.72	–	–	–	–	–	–

*OS, overall survival; DFS, disease-free survival; LCSS, lung cancer-specific survival; HR, hazard ratio; CI, confidence interval. When the HR > 1, the results supported the comparison group in front*.

We evaluated the possible factors that may affect survival of the Vein-first group vs. the Artery-first group for lobectomy. The results suggested that the advantages of survival in the Vein-First group were primarily embodied in the subgroups of SCC and earlier pathological TNM stage (I–II). For stage III NSCLC, no significant survival advantage was found in the Vein-first group, especially in the early published studies (Table 3).

**Table 3:** Subgroup analysis of survival (OS, DFS, and LCSS) in the comparison of Vein-first vs. Artery-first for lobectomy.

**Subgroups**	**No. of studies**	**Overall survival**		**No. of studies**	**Disease-free survival**		**No. of studies**	**Lung cancer-specific survival**	
		**HR (95% CI)**	***P***		**HR (95% CI)**	***P***		**HR (95% CI)**	***P***
**Total**	5	1.46 (1.12–1.91)	0.005	4	1.60 (1.23–2.08)	<0.001	2	1.64 (0.16–2.31)	0.005
**Published year**									
Earlier than 2016	2	0.99 (0.65–1.48)	0.94	1	0.76 (0.37–1.57)	0.46	1	1.26 (0.74–2.15)	0.4
2016–2020	3	1.96 (1.38–2.78)	<0.001	3	1.79 (1.35–2.37)	<0.001	1	1.98 (1.26–3.11)	0.003
**Country**									
China	3	1.69 (1.20–2.38)	0.003	3	1.51 (1.13–2.00)	0.004	1	1.98 (1.26–3.11)	0.003
Japan	1	1.79 (0.62–5.16)	0.28	1	2.36 (1.13–4.92)	0.02	–	–	–
Poland	1	1.08 (0.68–1.72)	0.75	–	–	–	1	1.26 (0.74–2.15)	0.4
**Tumor size, cm**									
≥3	1	1.94 (0.80–4.70)	0.14	1	1.61 (0.79–3.26)	0.19	–	–	–
Unrestricted	5	1.46 (1.12–1.91)	0.005	4	1.60 (1.23–2.08)	<0.001	–	–	–
**Histological type**									
Adenocarcinoma	1	1.54 (0.59–4.00)	0.37	1	1.14 (0.52–2.51)	0.75	–	–	–
Squamous cell carcinomas	1	4.00 (0.987–16.14)	0.052	1	3.01 (1.03–8.00)	0.04	–	–	–
Unrestricted (NSCLC)	5	1.46 (1.12–1.91)	0.005	4	1.60 (1.23–2.08)	<0.001	–	–	–
**Pathological TNM stage**									
I	1	2.06 (1.08–4.03)	0.04	2	1.65 (1.01–2.70)	0.05	1	2.14 (1.00–4.56)	0.05
II	1	3.39 (1.11–10.41)	0.03	1	2.63(1.01–6.86)	0.05	1	3.39 (1.11–10.41)	0.03
III	1	1.04 (0.54–2.00)	0.91	1	1.17 (0.69–2.00)	0.57	1	1.04 (0.54–2.00)	0.91
Unrestricted	5	1.46 (1.12–1.91)	0.005	4	1.60 (1.23–2.08)	<0.001	2	1.64 (0.16–2.31)	0.005
**Study design**									
RCT	1	1.08 (0.68–1.72)	0.75	4	1.60 (1.23–2.08)	<0.001	1	1.26 (0.74–2.15)	0.4
CT	4	1.70 (1.22–2.35)	0.002	–	–	–	1	1.98 (1.26–3.11)	0.003

*OS, overall survival; DFS, disease-free survival; LCSS, lung cancer-specific survival; HR, hazard ratio; CI, confidence interval; NSCLC, non-small cell lung cancer; RCT, randomized controlled trial; CT, cohort study; TNM, Tumor Node Metastasis. When the HR > 1, the results supported the Vein-First group*.

#### Intraoperative Indicators

Operative time (MD: −2.84, 95% CI: −24.70–19.02 min, *p* = 0.80, Supplementary Figure 4A), intraoperative blood loss (MD: 2.18, 95% CI: −19.41–23.78 min, *p* = 0.84, Supplementary Figure 4B), and blood transfusion (RR: 0.79, 95% CI: 0.41–1.54, *p* = 0.49, Supplementary Figure 4C) were similar between the two groups.

#### Hospitalization and Follow Up Indicators

Postoperative hospital stay (MD: 0.07, 95% CI: −0.32–0.45 days, *p* = 0.73, Supplementary Figure 5A), postoperative drainage time (MD: −0.07, 95% CI: −1.26–1.12 days, *p* = 0.91, Supplementary Figure 5B), total complications (RR: 1.15, 95% CI: 0.85–1.55, *p* = 0.35, Supplementary Figure 5C), total recurrences (RR: 0.89, 95% CI: 0.47–1.67, *p* = 0.71**)**, local recurrences (RR: 0.83, 95% CI: 0.33–2.13, *p* = 0.70**)**, and distant metastasis (RR: 0.76, 95% CI: 0.34–1.73, *p* = 0.52**)** were similar between the two groups (Supplementary Figure 6). Only one study (7) analyzed the CTCs and found that a higher rate of CTC increase (RR: 0.46, 95% CI: 0.27–0.79, *p* = 0.005, Supplementary Figure 7A), and a greater increase in CTCs was found in the Artery-first group (MD: −1.23, 95% CI: −1.86 to −0.60 Fu/3 ml, *p* = 0.0001, Supplementary Figure 7B).

#### Sensitivity Analysis

Significant heterogeneity was found in the analysis of operative time, intraoperative blood loss, and blood transfusion. Sensitivity analysis showed that removal of each study did not affect the stability or reliability of the results (Supplementary Figure 8).

#### Publication bias

No evidence of publication bias was found in the analysis of OS (Supplementary Figure 9A), DFS (Supplementary Figure 9B), and operative time (Supplementary Figure 9C).

## Discussion

With the increase in patients with NSCLC, standardization of the various details of surgical procedures to improve patient outcomes has become a hot research topic. The choice to first interrupt PA or PV during lobectomy is an important and easily neglected problem. Whether the Vein-first procedure can achieve better perioperative and survival outcomes compared with the Artery-first procedure is controversial (7–13). This study is the first meta-analysis to compare different vessel interruption sequences during lobectomy for a better clinical decision. The results suggested that the vein-first group had significantly better OS, DFS, and LCSS. The survival rates of OS at 2–5 years, DFS at 1–5 years, and LCSS at 3–5 years were also higher in the Vein-First group. Operative time, intraoperative blood loss, postoperative drainage time, total complications, and total recurrences were similar between the two groups.

Better survival was the greatest advantage for the Vein-first procedure compared to the Artery-first procedure. Similar results were also confirmed by Wei et al. (7). He et al. reported that the survival advantages of the Vein-first group were more significant for patients with SCC (8). The advantages of survival (OS, DFS, and LCSS) in the Vein-First group increased with the prolonged survival time. Two reasons might explain this advantage: (1) Once the effluent vein is blocked, tumor cells are less likely to enter the blood stream. Wei et al. reported that higher rates of incremental change in CTCs were observed in the Artery-first group (26/40 vs. 12/38, *P* = 0.003) (7). Higher expression levels of cancer-related indicators (CK19 mRNA, LUNX mRNA, pin1 mRNA, CD44v6, and CK19 genes) were also found in the Artery-first group after surgery than in the Vein-first group (21–23). (2) For most lung cancer surgeries, single-direction lobectomy with pulmonary vein ligation first may simplify the operational procedure, which decreases repeated grasping and manipulation of the tumor-bearing lobe during surgery (7). The expression levels of CD44v6 and CK19 were higher in the Artery-first group in the late period during surgery (22). Subgroup analyses suggested that the advantages of survival in the Vein-First group were primarily embodied in the subgroups of SCC and earlier pathological TNM stage (I–II). Similar survival outcomes between the two groups were reported by Li et al. (13) and Kozak et al. (10). Two reasons might explain this discrepancy: (1) A favorable trend had been found, but there was no statistical difference due to the small sample size in a single study (10). (2) The proportion of patients with stage I lung cancer was higher in Artery-first group (13). However, although efforts should be made to interrupt the pulmonary vein first for better oncologic results, tumor size and location may dictate an artery-first technique to ensure patient safety.

The main reason why some thoracic surgeons chose to interrupt the PA first is to reduce the risk of bleeding and loss of intravascular volume during surgery. However, the meta-analysis suggested that intraoperative blood loss and blood transfusion were similar between the two groups. Miller et al. reported that the PA blood flow of the lobe ceased almost immediately with the interruption of the PV (24). Wei et al. suggested that interrupting the PV first would not decrease unnecessary blood loss during surgery (7). Postoperative hospital stay, postoperative drainage time, and total complications were also similar between the two groups. For the follow-up of postoperative recurrence, we only found a trend favoring the Vein-first group without a significant difference, especially for distant metastasis. Sumitomo et al. reported that interrupting the PA first could significantly increase the risk of total recurrences and distant metastasis, which was consistent with our DFS data (HR: 1.60, 95% CI: 1.23–2.08, *p* < 0.001) (9). A significant increase in CTC count in drainage PV after surgical manipulation might be a reasonable explanation for the advantage of the Vein-first group (25). Taken together, interrupting the PV first may significantly decrease the risk of postoperative recurrence without increasing surgical risk.

However, several limitations must be mentioned. First, all of the included studies were published in English, which might introduce a language bias. Second, only two of the eight studies were RCTs, which decreased the quality of the data. Third, only 1,714 patients were included, which might reduce the credibility of the results. Fourth, seven of the eight studies were conducted in Asia. The results of our analysis might not be applicable to patients in other regions. Fifth, the follow-up time and surgical procedures were different between the included studies, which might increase the heterogeneity between studies. Sixth, the editions of TNM classification for pathological stage were different between the studies, which might affect the subgroup analyses according to the TNM classification.

## Conclusion

In summary, the Vein-first procedure appears to be the suitable choice of vessel interruption sequence during lobectomy for NSCLC with better survival (OS, DFS, and LCSS) and similar perioperative outcomes, especially for stage I–II SCC. The advantages of survival in the Vein-First group increased with prolonged survival. Due to the above limitations, the results must be confirmed in additional large sample RCTs. In complex lobectomy for NSCLC at special sites (e.g., tumor encroaching on the pulmonary vein), the sequence of vessel interruption must be determined according to the actual situation.

## Data Availability Statement

The original contributions presented in the study are included in the article/Supplementary Material, further inquiries can be directed to the corresponding author/s.

## Author Contributions

YL had full access to all of the data in the manuscript and takes responsibility for the integrity of the data and the accuracy of the data analysis. Drafting of the manuscript: XL, BW, and GL. Critical revision of the manuscript for important intellectual content: XL, BW, GL, DY, JP and YL. Statistical analysis: XL, GL, and YL. Supervision: WZ, XL, and YL. All authors. Concept and design, acquisition, analysis, or interpretation of data. All authors read and approved the final manuscript.

## Conflict of Interest

The authors declare that the research was conducted in the absence of any commercial or financial relationships that could be construed as a potential conflict of interest.

## Publisher's Note

All claims expressed in this article are solely those of the authors and do not necessarily represent those of their affiliated organizations, or those of the publisher, the editors and the reviewers. Any product that may be evaluated in this article, or claim that may be made by its manufacturer, is not guaranteed or endorsed by the publisher.

## Supplementary Material

Supplementary Figure 1 Comparisons of OSR (1–5 years) associated with Vein-first vs. Artery-first according to survival time.

Supplementary Figure 2 Comparisons of DFSR (1–5 years) associated with Vein-first vs. Artery-first according to survival time.

Supplementary Figure 3 Comparisons of LCSSR (1–5 years) associated with Vein-first vs. Artery-first according to survival time.

Supplementary Figure 4 Forest plots of intraoperative indicators: operative time **(A)**, intraoperative blood loss **(B)**, and blood transfusion **(C)**.

Supplementary Figure 5 Forest plots of hospitalization indicators: postoperative hospital stay **(A)**, postoperative drainage time **(B)**, and total complications **(C)**.

Supplementary Figure 6 Forest plots of follow up indicators: total recurrences **(A)**, local recurrences **(B)**, and distant metastasis **(C)**.

Supplementary Figure 7 Forest plots of CTCs after lobectomy: CTCs increase **(A)** and increment of CTCs **(B)**.

Supplementary Figure 8 Sensitivity analysis of operative time **(A)**, intraoperative blood loss **(B)**, and blood transfusion **(C)**.

Supplementary Figure 9 Publication bias of OS **(A)**, DFS **(B)**, and operative time **(C)**.

Supplementary Table 1 PRISMA 2009 Checklist.

Supplementary Table 2 Search strategy.

Supplementary Table 3 Methodological quality assessments of the included studies.

Supplementary Table 4 GRADE quality assessment by therapeutic strategy and study design for the outcomes.

## Abbreviations

NSCLC: non-small cell lung cancer
Vein-first: interrupting pulmonary vein first
Artery-first: interrupting pulmonary artery first
OS: overall survival
DFS: disease-free survival
LCSS: lung cancer-specific survival
OSR: overall survival rate
DFSR: disease-free survival rate
LCSSR: lung cancer-specific survival rate
HR: hazard ratio
SCC: squamous cell carcinoma
PA: pulmonary artery
PV: pulmonary vein
RCT: randomized clinical trial
SCC: squamous cell carcinoma
PRISMA: preferred reporting items for systematic reviews and meta-analyses
CTCs: circulating tumor cells
NOS: Newcastle-Ottawa scale
GRADE, grading of recommendations assessment, development: and evaluation
MD: difference in means
RR: risk ratio
CI: confidence interval
CT: cohort study
TNM: tumor node metastasis.
