# Supplementary figures and images for: Effects of Vessel Interruption Sequence During Lobectomy for Non-small Cell Lung Cancer: A Systematic Review and Meta-Analysis
Source: Front Surg. 2021 Jul 26;8:694005. doi: 10.3389/fsurg.2021.694005 (PMC8350043; doi:10.3389/fsurg.2021.694005)

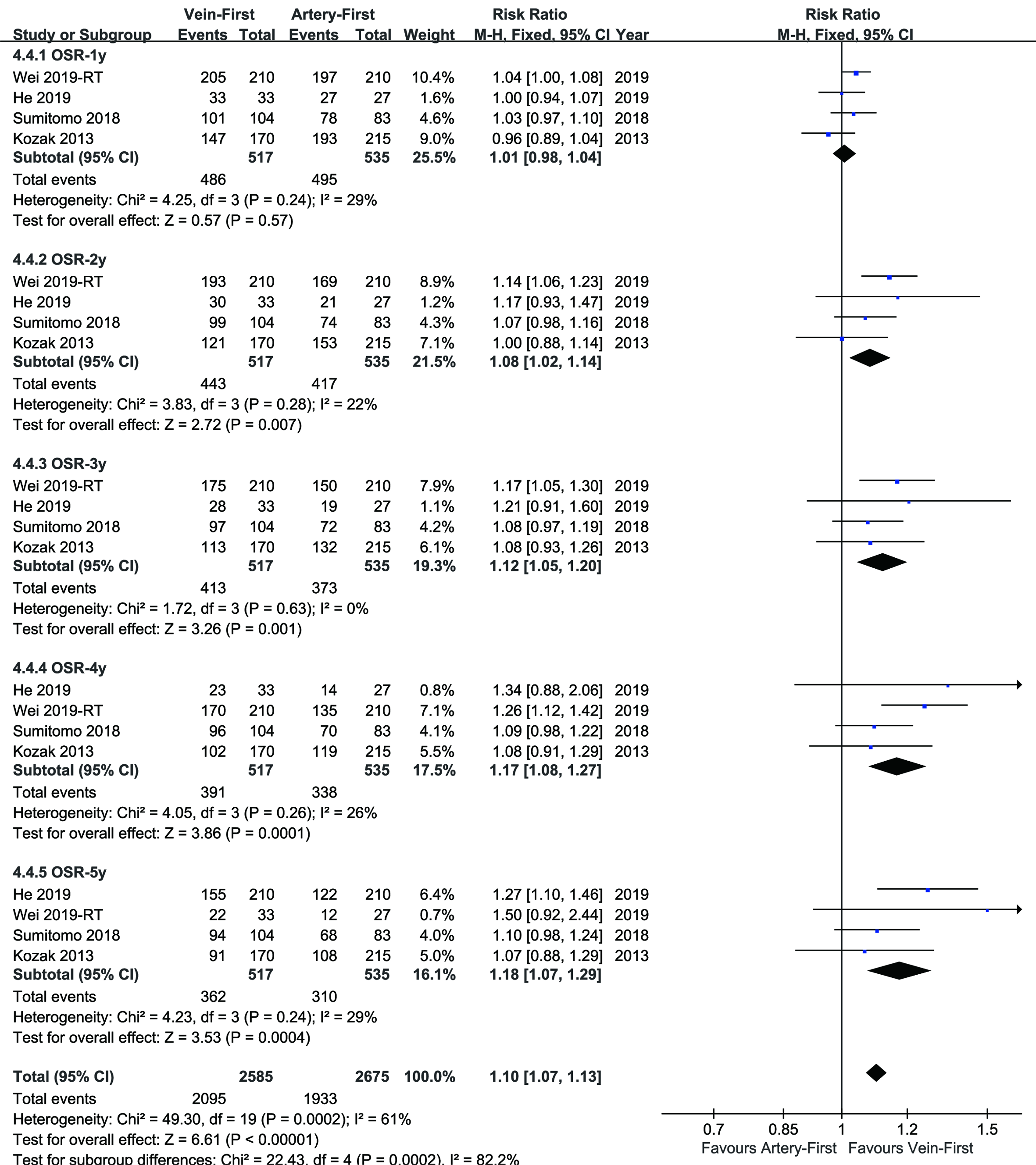

Supplement: Supplementary Figure 1 — Comparisons of OSR (1–5 years) associated with Vein-first vs. Artery-first according to survival time. [file Image_1.TIF]

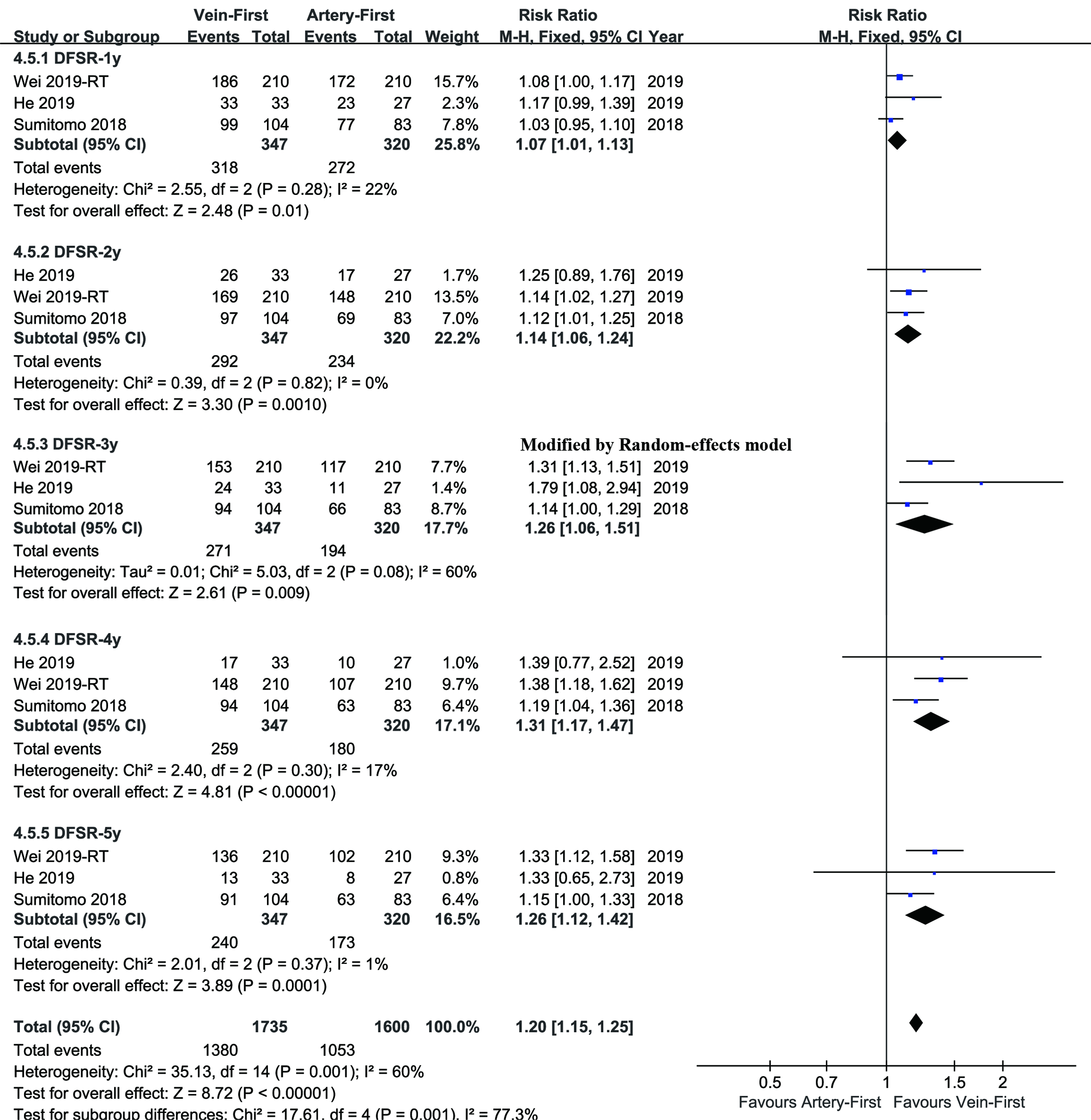

Supplement: Supplementary Figure 2 — Comparisons of DFSR (1–5 years) associated with Vein-first vs. Artery-first according to survival time. [file Image_2.TIF]

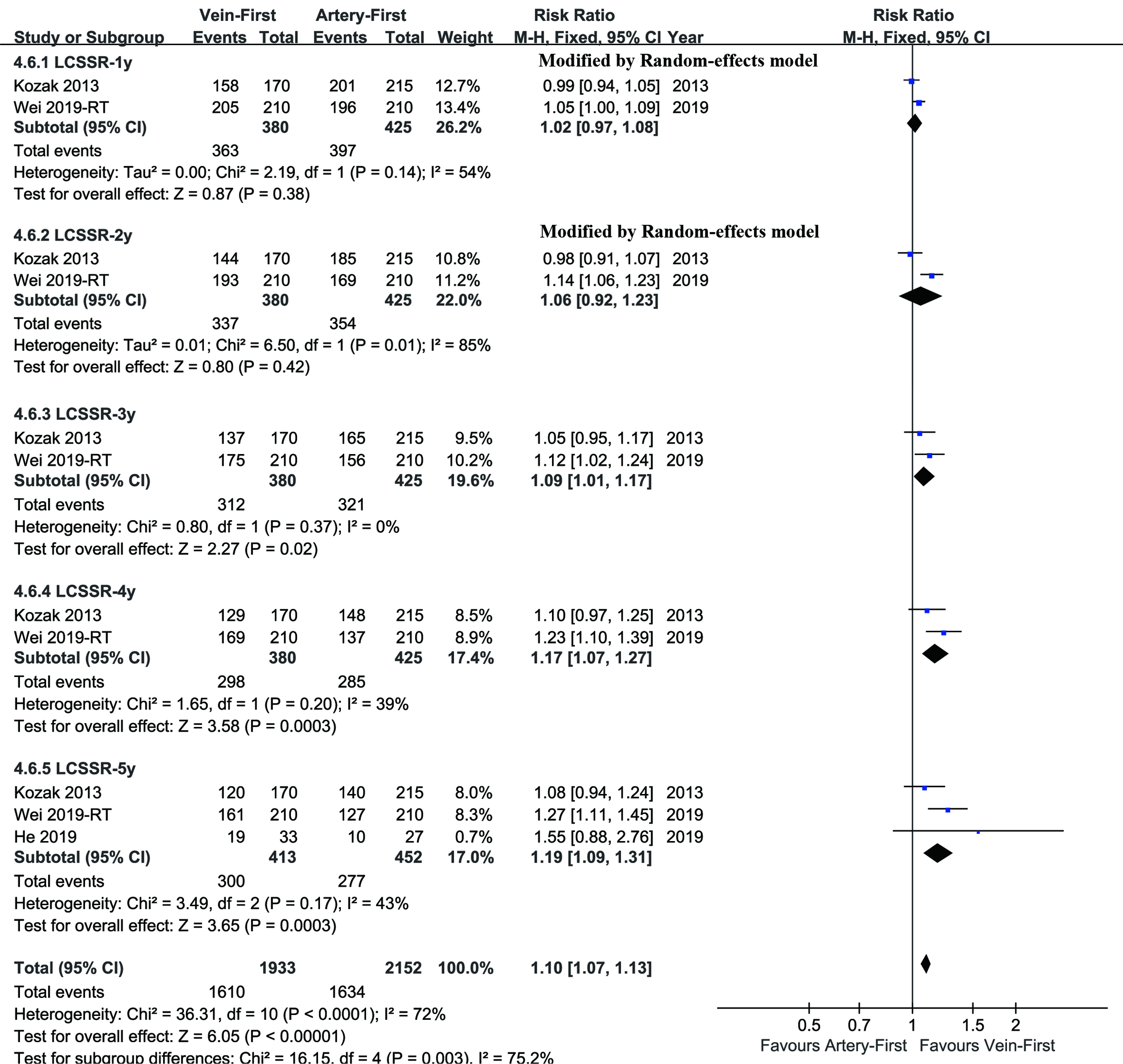

Supplement: Supplementary Figure 3 — Comparisons of LCSSR (1–5 years) associated with Vein-first vs. Artery-first according to survival time. [file Image_3.TIF]

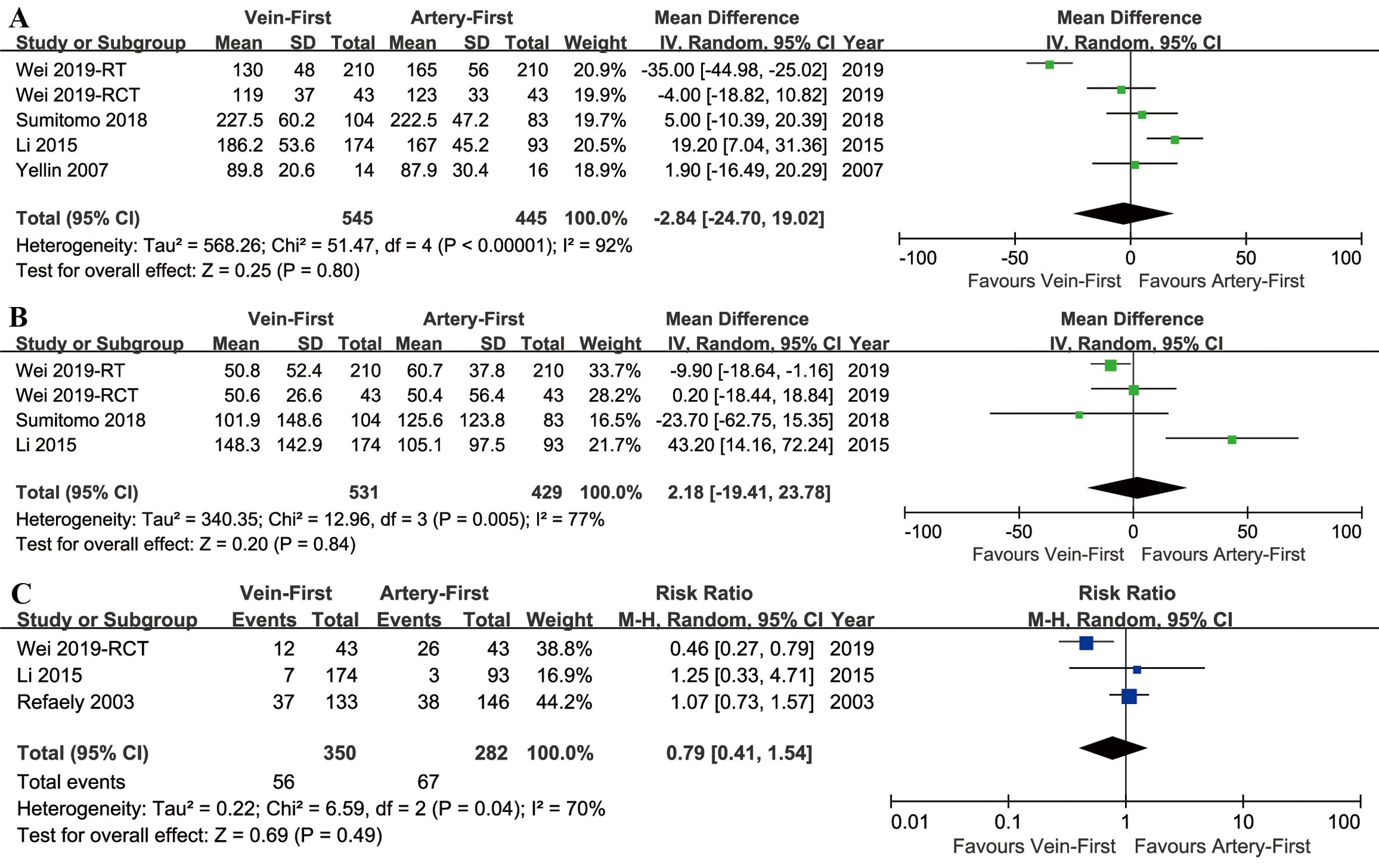

Supplement: Supplementary Figure 4 — Forest plots of intraoperative indicators: operative time (A), intraoperative blood loss (B), and blood transfusion (C). [file Image_4.TIF]

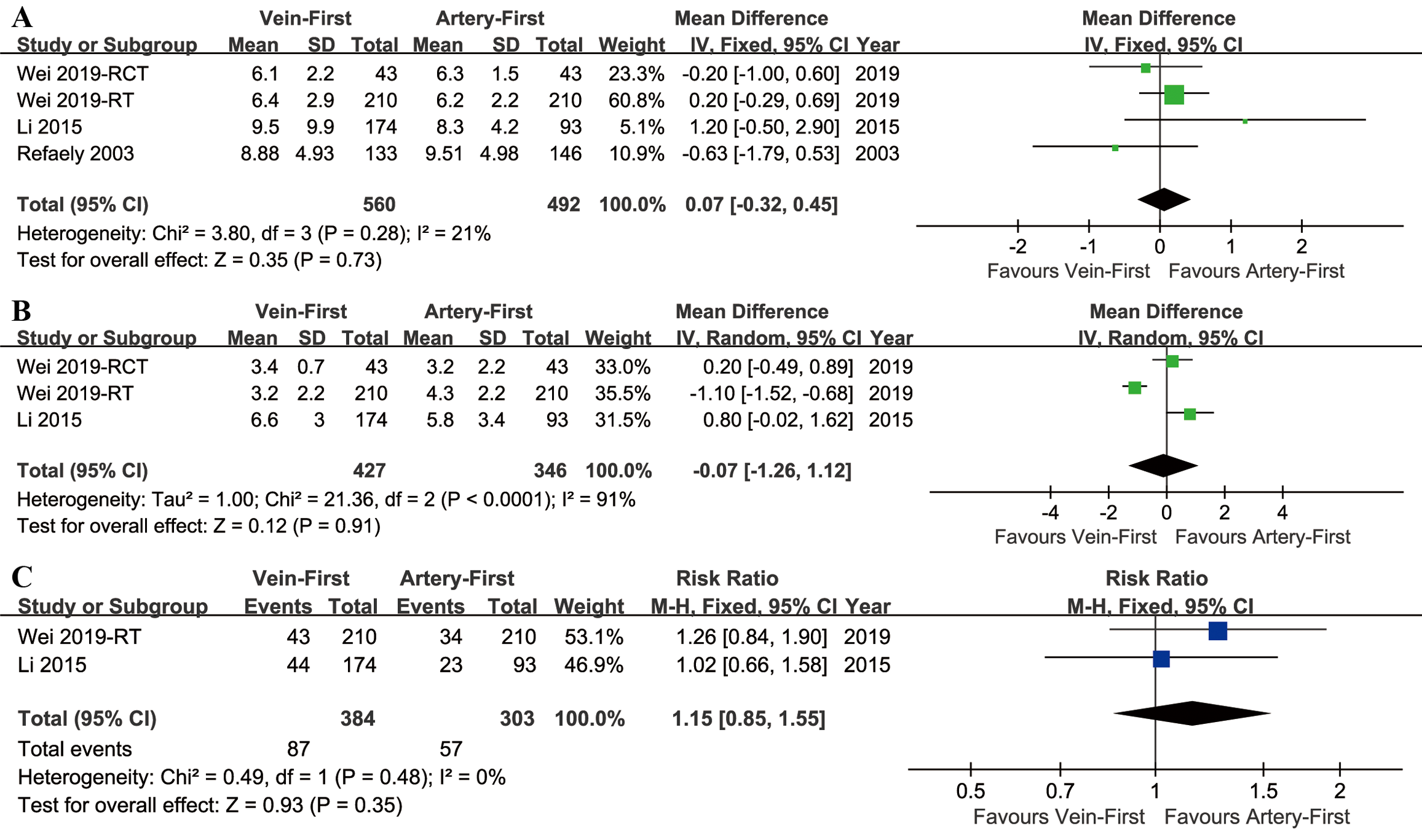

Supplement: Supplementary Figure 5 — Forest plots of hospitalization indicators: postoperative hospital stay (A), postoperative drainage time (B), and total complications (C). [file Image_5.TIF]

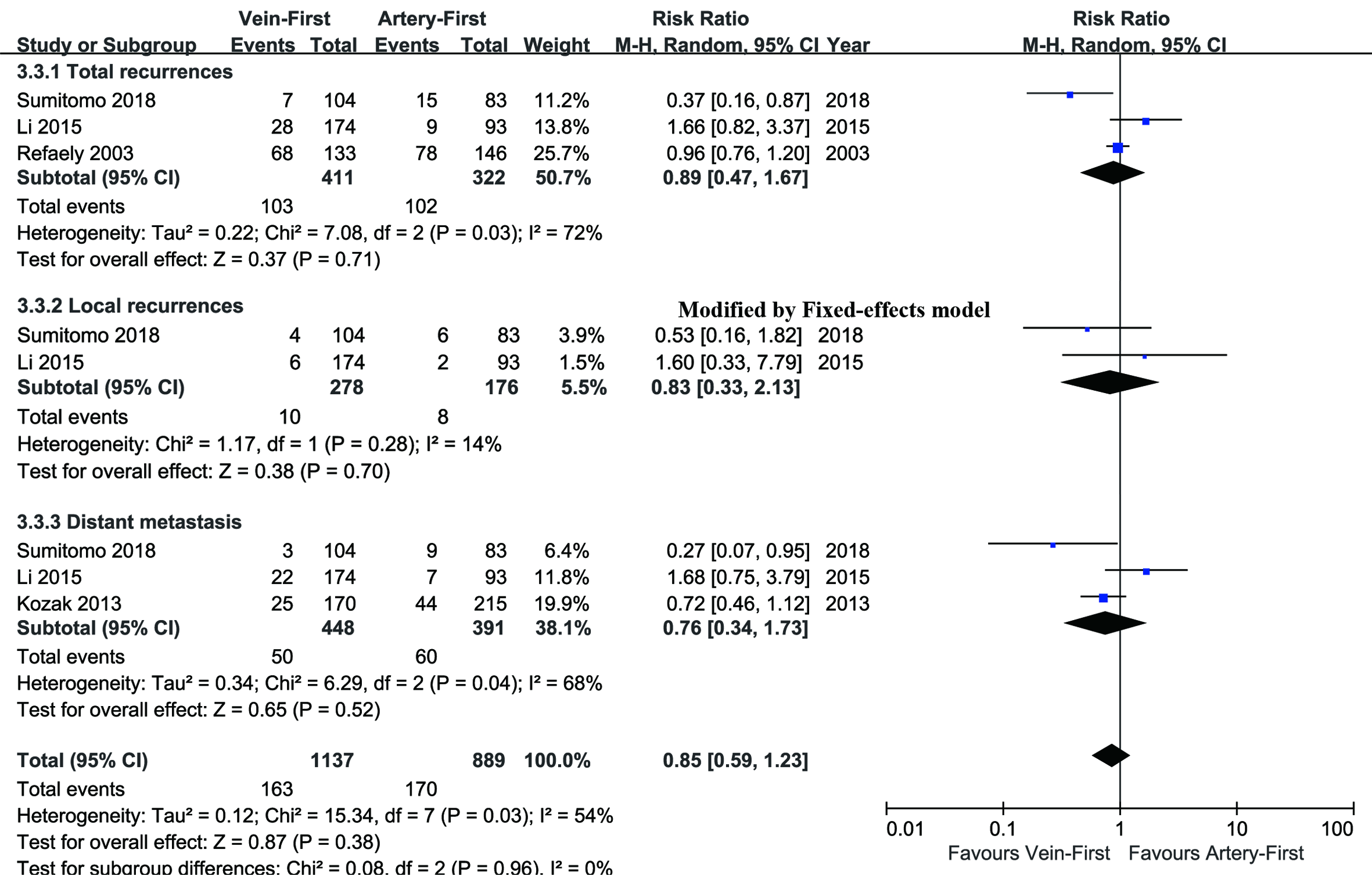

Supplement: Supplementary Figure 6 — Forest plots of follow up indicators: total recurrences (A), local recurrences (B), and distant metastasis (C). [file Image_6.TIF]

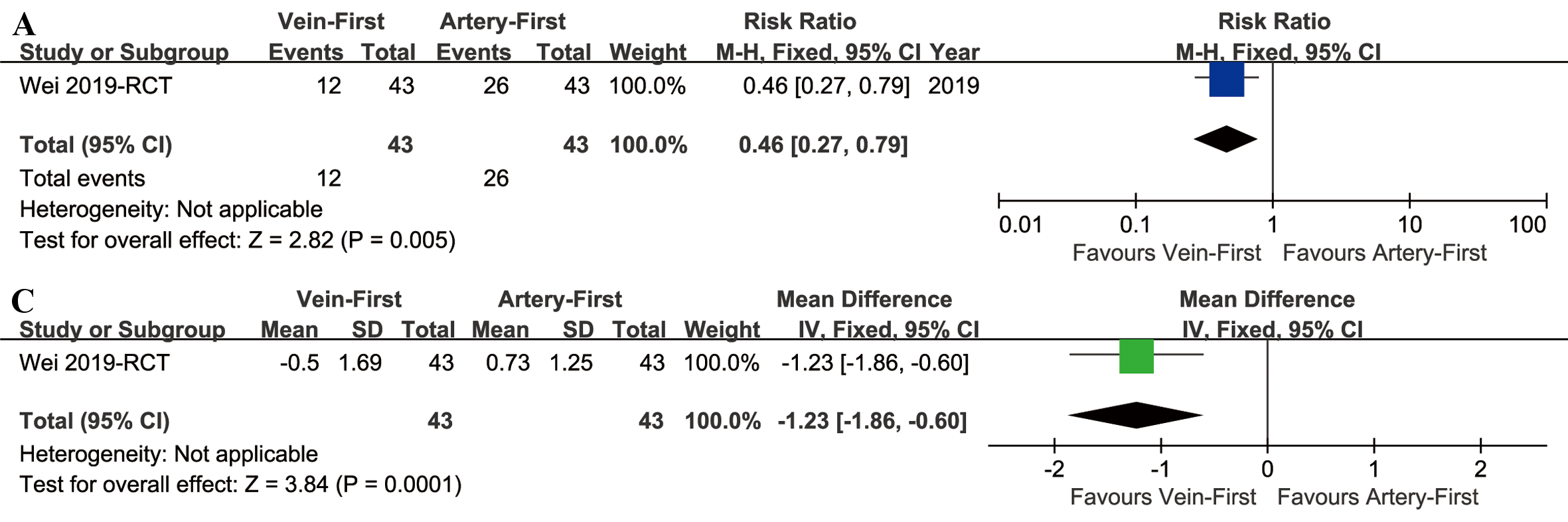

Supplement: Supplementary Figure 7 — Forest plots of CTCs after lobectomy: CTCs increase (A) and increment of CTCs (B). [file Image_7.TIF]

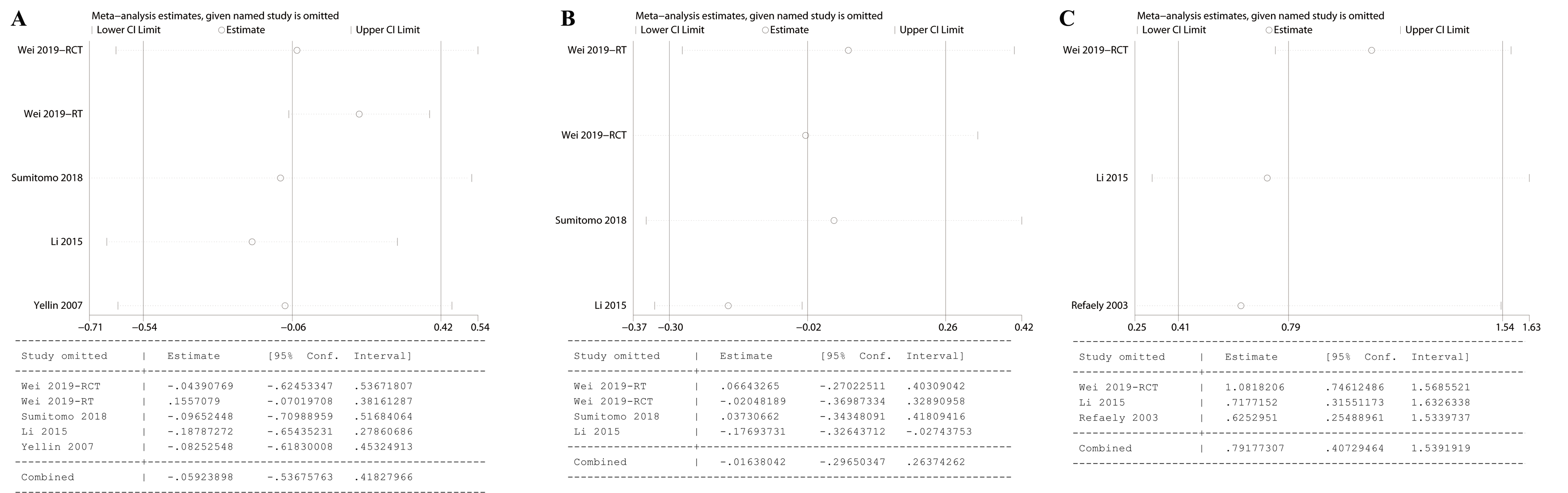

Supplement: Supplementary Figure 8 — Sensitivity analysis of operative time (A), intraoperative blood loss (B), and blood transfusion (C). [file Image_8.TIF]

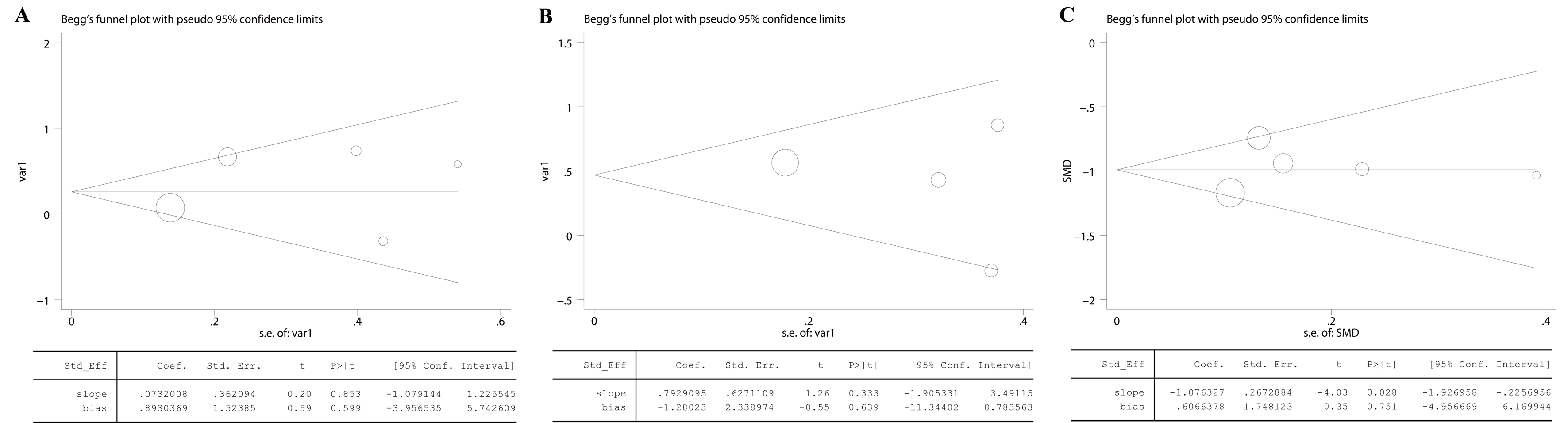

Supplement: Supplementary Figure 9 — Publication bias of OS (A), DFS (B), and operative time (C). [file Image_9.TIF]
