# Supplementary material for: Effects of Vessel Interruption Sequence During Lobectomy for Non-small Cell Lung Cancer: A Systematic Review and Meta-Analysis
Source: Front Surg. 2021 Jul 26;8:694005. doi: 10.3389/fsurg.2021.694005 (PMC8350043; doi:10.3389/fsurg.2021.694005)
Supplement: Supplementary Table 2 — Search strategy. [file Table_2.DOCX]

**Table S2** Search strategy

| **PubMed**  The database was searched on December 5, 2020, n=175.  Search Strategy:  (Vein[Title/Abstract]) AND (Artery[Title/Abstract]) AND (Lung neoplasms[Title/Abstract] OR Pulmonary Neoplasms[Title/Abstract] OR Neoplasms, Lung[Title/Abstract] OR Lung Neoplasm[Title/Abstract] OR Neoplasm, Lung[Title/Abstract] OR Neoplasms, Pulmonary[Title/Abstract] OR Neoplasm, Pulmonary[Title/Abstract] OR Pulmonary Neoplasm[Title/Abstract] OR Lung Cancer[Title/Abstract] OR Cancer, Lung[Title/Abstract] OR Cancers, Lung[Title/Abstract] OR Lung Cancers[Title/Abstract] OR Pulmonary Cancer[Title/Abstract] OR Cancer, Pulmonary[Title/Abstract] OR Cancers, Pulmonary[Title/Abstract] OR Pulmonary Cancers[Title/Abstract] OR Cancer of the Lung[Title/Abstract] OR Cancer of Lung[Title/Abstract]) |
| --- |
| **Web of Science**  The database was searched on December 5, 2020, n=334.  Search Strategy:  1 TOPIC: (“Vein”) (20703)  2 TOPIC: (“Artery”) (20703)  3 TOPIC: (“Lung neoplasms” OR “Pulmonary Neoplasms” OR “Neoplasms, Lung” OR “Lung Neoplasm” OR “Neoplasm, Lung” OR “Neoplasms, Pulmonary” OR “Neoplasm, Pulmonary” OR “Pulmonary Neoplasm” OR “Lung Cancer” OR “Cancer, Lung” OR “Cancers, Lung” OR “Lung Cancers” OR “Pulmonary Cancer” OR “Cancer, Pulmonary” OR “Cancers, Pulmonary” OR “Pulmonary Cancers” OR “Cancer of the Lung” OR “Cancer of Lung”) (15603)  4 #1 AND #2 AND #3 (1104) |
| **EMBASE**  The database was searched on December 5, 2020, n=441.  Search Strategy:  ('Vein':ti,ab,kw) AND ('Artery':ti,ab,kw) AND ('Lung neoplasms':ti,ab,kw OR 'Pulmonary Neoplasms':ti,ab,kw OR 'Neoplasms, Lung':ti,ab,kw OR 'Lung Neoplasm':ti,ab,kw OR 'Neoplasm, Lung':ti,ab,kw OR 'Neoplasms, Pulmonary':ti,ab,kw OR 'Neoplasm, Pulmonary':ti,ab,kw OR 'Pulmonary Neoplasm':ti,ab,kw OR 'Lung Cancer':ti,ab,kw OR 'Cancer, Lung':ti,ab,kw OR 'Cancers, Lung':ti,ab,kw OR 'Lung Cancers':ti,ab,kw OR 'Pulmonary Cancer':ti,ab,kw OR 'Cancer, Pulmonary':ti,ab,kw OR 'Cancers, Pulmonary':ti,ab,kw OR 'Pulmonary Cancers':ti,ab,kw OR 'Cancer of the Lung':ti,ab,kw OR 'Cancer of Lung':ti,ab,kw) |
| **Cochrane Library**  The database was searched on December 5, 2020, n=99.  Search Strategy:  (“Vein”): ti,ab,kw AND (“Artery”): ti,ab,kw AND (“Lung neoplasms” OR “Pulmonary Neoplasms” OR “Neoplasms, Lung” OR “Lung Neoplasm” OR “Neoplasm, Lung” OR “Neoplasms, Pulmonary” OR “Neoplasm, Pulmonary” OR “Pulmonary Neoplasm” OR “Lung Cancer” OR “Cancer, Lung” OR “Cancers, Lung” OR “Lung Cancers” OR “Pulmonary Cancer” OR “Cancer, Pulmonary” OR “Cancers, Pulmonary” OR “Pulmonary Cancers” OR “Cancer of the Lung” OR “Cancer of Lung”): ti,ab,kw - (Word variations have been searched) |
| **Ovid MEDLINE**  The database was searched on December 5, 2020, n=1258.  Search Strategy:  1 Vein.ab. (226987)  2 Artery.ab. (438191)  3 Pulmonary Neoplasms.ab. (1168)  4 Neoplasms, Lung.ab. (625)  5 Lung Neoplasm.ab. (1467)  6 Neoplasm, Lung.ab. (313)  7 Neoplasms, Pulmonary.ab. (128)  8 Neoplasm, Pulmonary.ab. (143)  9 Pulmonary Neoplasm.ab. (796)  10 Lung Cancer.ab. (337884)  11 Cancer, Lung.ab. (13998)  12 Cancers, Lung.ab. (5587)  13 Lung Cancers.ab. (36897)  14 Pulmonary Cancer.ab. (1875)  15 Cancer, Pulmonary.ab. (2433)  16 Cancers, Pulmonary.ab. (210)  17 Pulmonary Cancers.ab. (363)  18 Cancer of the Lung.ab. (21577)  19 Cancer of Lung.ab. (16519)  20 or/3-19 [Lung cancer] (467886)  21 1 and 2 and 20 (1258) |
| **ScienceDirect**  The database was searched on December 5, 2020, n=62.  Search Strategy:  Title, abstract, keywords: ((“Vein”) and (“Artery”) and (“Lung neoplasms” OR “Pulmonary Neoplasms” OR “Neoplasms, Lung” OR “Lung Neoplasm” OR “Neoplasm, Lung” OR “Neoplasms, Pulmonary” OR “Neoplasm, Pulmonary” OR “Pulmonary Neoplasm” OR “Lung Cancer” OR “Cancer, Lung” OR “Cancers, Lung” OR “Lung Cancers” OR “Pulmonary Cancer” OR “Cancer, Pulmonary” OR “Cancers, Pulmonary” OR “Pulmonary Cancers” OR “Cancer of the Lung” OR “Cancer of Lung”)) |
| **Scopus**  The database was searched on December 5, 2020, n=2024.  Search Strategy:  TITLE-ABS-KEY ((“Vein”) and (“Artery”) and (“Lung neoplasms” OR “Pulmonary Neoplasms” OR “Neoplasms, Lung” OR “Lung Neoplasm” OR “Neoplasm, Lung” OR “Neoplasms, Pulmonary” OR “Neoplasm, Pulmonary” OR “Pulmonary Neoplasm” OR “Lung Cancer” OR “Cancer, Lung” OR “Cancers, Lung” OR “Lung Cancers” OR “Pulmonary Cancer” OR “Cancer, Pulmonary” OR “Cancers, Pulmonary” OR “Pulmonary Cancers” OR “Cancer of the Lung” OR “Cancer of Lung”)) |

**Note:** The combined text and medical subject heading (MeSH) terms used were: “Vein”, “Artery” and “Lung cancer”.
