# Supplementary material for: Effects of Vessel Interruption Sequence During Lobectomy for Non-small Cell Lung Cancer: A Systematic Review and Meta-Analysis
Source: Front Surg. 2021 Jul 26;8:694005. doi: 10.3389/fsurg.2021.694005 (PMC8350043; doi:10.3389/fsurg.2021.694005)
Supplement: Supplementary Table 3 — Methodological quality assessments of the included studies. [file Table_3.DOC]

**Table S3 Methodological quality assessments of the included studies.**

| **Study** | | Randomization | Masking | Accountability of all patients | Selection | | | | Comparability^d^ | Outcome | | | Total score |
| --- | --- | --- | --- | --- | --- | --- | --- | --- | --- | --- | --- | --- | --- |
|  |  |  |  |  | Exposed cohort^a^ | Nonexposed cohort^b^ | Ascertainment of exposure | Outcome of interest^c^ |  | Assessment of outcome | Length of follow-up^e^ | Adequacy of follow-up |  |
| 2019 | Wei [7]-RCT | ** | ** | * |  |  |  |  |  |  |  |  | 5 |
| 2013 | Kozak [10] | ** | * | * |  |  |  |  |  |  |  |  | 4 |
| 2019 | Wei [7]-RT |  |  |  | * | * | * | * | ** | * | * | * | 9 |
| 2019 | He [8] |  |  |  | * |  | * | * | ** |  | * | * | 7 |
| 2018 | Sumitomo [9] |  |  |  | * |  | * | * | ** | * |  | * | 7 |
| 2015 | Li [13] |  |  |  | * | * | * | * | ** | * | * | * | 9 |
| 2007 | Yellin [11] |  |  |  | * | * |  | * | ** | * |  | * | 7 |
| 2003 | Refaely [12] |  |  |  | * | * | * | * | ** | * |  | * | 8 |

Note: ^a^ Representativeness of the exposed cohort;

^b^ Selection of the non-exposed cohort;

^c^ Demonstration that outcome of interest was not present at start of study;

^d^ Comparability of cohorts on the basis of the design or analysis;

^e^ Was follow-up long enough for outcomes to occur.
