# Supplementary material for: Effects of Vessel Interruption Sequence During Lobectomy for Non-small Cell Lung Cancer: A Systematic Review and Meta-Analysis
Source: Front Surg. 2021 Jul 26;8:694005. doi: 10.3389/fsurg.2021.694005 (PMC8350043; doi:10.3389/fsurg.2021.694005)
Supplement: Supplementary Table 4 — GRADE quality assessment by therapeutic strategy and study design for the outcomes. [file Table_4.DOC]

**Table S4** GRADE quality assessment by therapeutic strategy and study design for the outcomes.

| **Primary outcomes** | **No. of Studies** | **No. of Participants** | | **Differences (95%CI) ^a^** | **Quality Assessment** | | | | | **Quality** |
| --- | --- | --- | --- | --- | --- | --- | --- | --- | --- | --- |
|  |  | **Vein-First** | **Artery-First** |  | **Risk of Bias ^b^** | **Inconsistency** | **Indirectness** | **Imprecision** | **Publication Bias ^c^** |  |
| **Survival** |  |  |  |  |  |  |  |  |  |  |
| OS | 5 | 691 | 628 | 1.46 [1.12, 1.91] | Serious (-1) | No inconsistency | No indirectness | No imprecision | Unlikely | Very Low |
| OSR |  |  |  |  |  |  |  |  |  |  |
| 1-year | 4 | 486/517 | 495/535 | 1.01 [0.98, 1.04] | Serious (-1) | No inconsistency | No indirectness | No imprecision | Unlikely | Very Low |
| 2-year | 4 | 443/517 | 417/535 | 1.08 [1.02, 1.14] | Low | No inconsistency | No indirectness | No imprecision | Unlikely | Low |
| 3-year | 4 | 413/517 | 373/535 | 1.12 [1.05, 1.20] | Low | No inconsistency | No indirectness | No imprecision | Unlikely | Low |
| 4-year | 4 | 391/517 | 338/535 | 1.17 [1.08, 1.27] | Low | No inconsistency | No indirectness | No imprecision | Unlikely | Low |
| 5-year | 4 | 362/517 | 310/535 | 1.18 [1.07, 1.29] | Low | No inconsistency | No indirectness | No imprecision | Unlikely | Low |
| DFS | 4 | 521 | 413 | 1.60 [1.23, 2.08] | Low | No inconsistency | No indirectness | No imprecision | Unlikely | Low |
| DFSR |  |  |  |  |  |  |  |  |  |  |
| 1-year | 4 | 318/347 | 272/320 | 1.07 [1.01, 1.13] | Low | No inconsistency | No indirectness | No imprecision | Unlikely | Low |
| 2-year | 4 | 292/347 | 234/320 | 1.14 [1.06, 1.24] | Low | No inconsistency | No indirectness | No imprecision | Unlikely | Low |
| 3-year | 4 | 271/347 | 194/320 | 1.26 [1.06, 1.51] | Low | No inconsistency | No indirectness | No imprecision | Unlikely | Low |
| 4-year | 4 | 259/347 | 180/320 | 1.31 [1.17, 1.47] | Low | No inconsistency | No indirectness | No imprecision | Unlikely | Low |
| 5-year | 4 | 240/347 | 173/320 | 1.26 [1.12, 1.42] | Low | Serious (-1) | No indirectness | No imprecision | Unlikely | Very Low |
| LCSS | 2 | 380 | 425 | 1.64 [1.16, 2.31] | Low | No inconsistency | No indirectness | No imprecision | Unlikely | Low |
| LCSSR |  |  |  |  |  |  |  |  |  |  |
| 1-year | 2 | 363/380 | 397/425 | 1.02 [0.97, 1.08] | Low | No inconsistency | No indirectness | No imprecision | Unlikely | Low |
| 2-year | 2 | 337/380 | 354/425 | 1.06 [0.92, 1.23] | Low | No inconsistency | No indirectness | No imprecision | Unlikely | Low |
| 3-year | 2 | 312/380 | 321/425 | 1.09 [1.01, 1.17] | Low | No inconsistency | No indirectness | No imprecision | Unlikely | Low |
| 4-year | 2 | 298/380 | 285/425 | 1.17 [1.07, 1.27] | Low | No inconsistency | No indirectness | No imprecision | Unlikely | Low |
| 5-year | 3 | 300/413 | 277/452 | 1.19 [1.09, 1.31] | Low | No inconsistency | No indirectness | No imprecision | Unlikely | Low |
| **Operative outcomes** |  |  |  |  |  |  |  |  |  |  |
| Operative time | 5 | 545 | 445 | -2.84 [-24.70, 19.02] | Very Serious (-2) | Very Serious (-2) | No indirectness | No imprecision | Unlikely | Very Low |
| Intraoperative blood loss | 4 | 531 | 429 | 2.18 [-19.41, 23.78] | Very Serious (-2) | Very Serious (-2) | No indirectness | Serious (-1) | Unlikely | Very Low |
| Blood transfusion | 3 | 56/350 | 67/282 | 0.78 [0.41, 1.54] | Unclear | Very Serious (-2) | No indirectness | No imprecision | Unlikely | Very Low |
| **Hospitalization and follow up outcomes** | |  |  |  |  |  |  |  |  |  |
| Postoperative hospital stay | 4 | 560 | 492 | 0.07 [-0.32, 0.45] | Low | No inconsistency | No indirectness | No imprecision | Unlikely | Low |
| Postoperative drainage time | 3 | 427 | 348 | -0.07 [-1.28, 1.12] | Low | No inconsistency | No indirectness | No imprecision | Unlikely | Low |
| Total complications | 2 | 87/384 | 57/303 | 1.15 [0.85, 1.55] | Low | No inconsistency | No indirectness | No imprecision | Unlikely | Low |
| Patients with CTCs increase | 1 | 12/43 | 26/43 | 0.46 [0.27, 0.79] | Low | No inconsistency | No indirectness | No imprecision | Unlikely | Low |
| Increment of CTCs | 1 | 43 | 43 | -1.23 [-1.86, -0.60] | Low | Serious (-1) | No indirectness | No imprecision | Unlikely | Very Low |
| Total recurrences | 3 | 103/411 | 102/322 | 0.89 [0.47, 1.67] | Low | No inconsistency | No indirectness | No imprecision | Unlikely | Low |
| Local recurrences | 2 | 10/278 | 8/176 | 0.83 [0.33, 2.13] | Low | No inconsistency | No indirectness | No imprecision | Unlikely | Low |
| Distant metastasis | 3 | 50/448 | 60/391 | 0.76 [0.34, 1.73] | Low | No inconsistency | No indirectness | No imprecision | Unlikely | Low |

**Abbreviations:** OS: overall survival; OSR: overall survival rate; DFS: disease-free survival; DFSR: disease-free survival rate; LCSS: lung cancer-specific survival; LCSSR: lung cancer-specific survival rate; CTCs: circulating tumor cells; CI: confidence interval.

^a^ Differences: hazard ratio (HR) for OS, DFS and LCSSS; risk ratios (RR) for OSR, DFSR, LCSSR, blood transfusion, total complications, patients with CTCs increase, total recurrences, local recurrences, distant metastasis; mean difference (MD) for operative time, intraoperative blood loss, postoperative hospital stay, postoperative drainage time and Increment of CTCs.

^b^ Risk of bias assessed using the Newcastle-Ottawa Scale (NOS) for non-randomized studies and Jadad scale for randomized controlled trials.

^c^ Publication bias was assessed by Egger’s and Begg’s tests.
